# Arrhythmias and clinical outcomes in Fabry disease with cardiac and renal involvement

**DOI:** 10.1186/s13023-025-04079-3

**Published:** 2025-11-05

**Authors:** Xiang Yin, Zhuonan Song, Xiangjie Sun, Xiaogang Guo, Tianxin Ye, Fangcong Yu, Hui Yan, Xiaosheng Hu

**Affiliations:** Department of Cardiology, The First Affiliated Hospital, School of Medicine, Zhejiang University, NO. 79 Qingchun Road, Hangzhou, Zhejiang Province 310003 P.R. China

**Keywords:** Fabry disease, Cardiomyopathy, Nephropathy, Arrhythmia, Clinical outcomes

## Abstract

Both cardiomyopathy and nephropathy represent hallmark manifestations of Fabry disease (FD), evidence remains limited regarding cardiovascular risk stratification based on organ-specific involvement patterns. In this retrospective cohort study of FD patients from the First Affiliated Hospital of Zhejiang University, we evaluated associations between cardiac/renal involvement and major cardiovascular events, including: (1) cardiovascular mortality, (2) new-onset severe heart failure, (3) incident atrial fibrillation, and (4) significant arrhythmia requiring device implantation. Patients were stratified into four phenotypic groups: non-affected (24.1%, *n* = 20), cardiac-only (24.1%, *n* = 20), renal-only (18.1%, *n* = 15), and co-affected (33.7%, *n* = 28). During a median follow-up of 39 months, 36.1% (*n* = 30) reached the primary endpoint. The cohort demonstrated substantial arrhythmia burden with significant intergroup differences (*p* = 0.028). Univariate Cox analysis revealed both left ventricular maximum wall thickness (LVMWT; HR: 1.176, 95% CI: 1.050–1.336, *p* = 0.010) and estimated glomerular filtration rate (eGFR; HR: 0.978, 95% CI: 0.962–0.992, *p* < 0.001) as continuous variables were significantly associated with outcomes. However, multivariate analysis confirmed only LVMWT remained an independent predictor (*p* = 0.010), while eGFR lost its significance (*p* = 0.100). These findings demonstrate that organ involvement patterns exert a critical influence on cardiovascular prognosis in patients with FD, providing a framework for refined risk stratification.

**Supplementary Information:**

The online version contains supplementary material available at 10.1186/s13023-025-04079-3.

## Introduction

Fabry disease (FD) is an X-linked lysosomal storage disorder caused by α-galactosidase A (α-GalA) deficiency, leading to systemic accumulation of lysosomal globotriaosylceramide (Gb-3/GL-3) [1, 2]. This accumulation disrupts cellular homeostasis, impairing critical processes including signaling, energy metabolism, and autophagy, whilst simultaneously triggering chronic inflammation and fibrosis [3]. These pathological changes ultimately lead to multi-organ dysfunction, particularly affecting cardiac and renal systems [4].

As a pan-ethnic disease, the prevalence of FD far exceeds initial estimates [4–6]. Registry data indicate that left ventricular hypertrophy (LVH; develops in 53% of males and ≥ 33% of females) and chronic kidney disease (CKD; develops in 28% males, 13% females) are more common among patients with FD [5, 7, 8]. Concurrently, early cardiac electrical remodelling and the subsequent development of arrhythmias are also frequently observed in Fabry disease [9, 10]. Recent evidence highlights arrhythmias—including conduction defects, atrial fibrillation (AF), and ventricular arrhythmias—as key drivers of FD-related morbidity and mortality, even sudden cardiac death (SCD) [11, 12]. Despite these well-characterized organ-specific complications, no existing studies have systematically evaluated prognosis based on distinct patterns of cardiac versus renal involvement. Therefore, this study aims to quantify arrhythmia prevalence across different FD phenotypic subgroups and assess the prognostic significance of organ-specific injury patterns.

## Methods

This single-centre retrospective study screened 113 patients with FD at the First Affiliated Hospital of Zhejiang University School of Medicine between January 2020 and December 2024, ultimately enrolling 83 cases. A flow diagram of patient inclusion/exclusion was shown in Supplementary Fig. 1. Exclusion criteria comprised missing or incomplete echocardiography or electrocardiogram (ECG) evaluations (*n* = 20) and unavailable/lost-to-follow-up cases (*n* = 10). For each enrolled patient, the initial electronic echocardiogram records and 12-lead ECG findings were reviewed to extract clinical data. Additional evaluations, such as cardiac/renal biopsy, cardiac magnetic resonance (CMR) imaging, Holter monitoring, renal ultrasound, and 24-hour urine protein measurements were collected when available.

FD diagnosis was established through plasma and leucocyte alpha-galactosidase A enzyme activity (for male patients) and confirmed by sequencing of the GLA gene (for all patients) [5]. Genetic factors and pathological biopsies were considered when available. Final diagnoses were confirmed by a panel of experts specializing in Fabry disease.

### Organ involvement assessment

According to previous research [13, 14], cardiac involvement was defined by echocardiography or CMR with the following parameters:1) Left ventricular (LV) maximal wall thickness >12 mm;2) Left atrial volume index (LAVi) >34 mL/m²;3) LV ejection fraction (LVEF) < 50%;4) Tricuspid annular plane systolic excursion (TAPSE) <17 mm.

Renal Involvement was defined by any of the following [8, 15]:1) Proteinuria >300 mg/24 h;2) Estimated glomerular filtration rate (eGFR) below the age-specific 2nd percentile;3) Chronic kidney disease (CKD) evidence on renal ultrasound.4) Pathological confirmation via renal biopsy.

Patients were stratified into four groups based on organ involvement:

#### non-affected

No evidence of cardiac or renal structural involvement.

#### cardiac-only

Echocardiographic abnormalities were observed without renal pathology.

#### renal-only

Renal involvement was confirmed without echocardiographic abnormalities.

#### co-affected

Both cardiac and renal involvement confirmed.

### Electrophysiological data collection and analysis

Electrophysiological parameters were systematically collected, including baseline heart rate (HR), PR interval, QRS duration, and corrected QT interval (QTc, calculated using Bazett’s formula [16]). Two blinded cardiologists independently analysed all tracings to identify conduction abnormalities, voltage changes, and arrhythmias without access to clinical data.

### Study endpoints

Regular clinical evaluations occurred at 6–24 months intervals (or earlier if clinically indicated). Primary endpoints included:1) Cardiovascular mortality;2) New-onset severe heart failure (New York Heart Association (NYHA) class III/IV);3) Incident AF;4) Significant bradyarrhythmias or tachyarrhythmias requiring device implantation (pacemaker or implantable cardioverter-defibrillator (ICD)).

Patients were followed through Dec 2024 via clinical evaluations at the referral centre (outpatient visits or hospital admissions) and structured telephone interviews (when in-person follow-up was unavailable).

The study protocol received approval from the institutional ethics committees of the First Affiliated Hospital, Zhejiang University School of Medicine and was conducted in compliance with the most recent revision of the Helsinki Declaration.

### Statistical analysis

Continuous variables are presented as median (interquartile range) and analysed using the Kruskal-Wallis test. Categorical variables are expressed as counts (percentages), with between-group comparisons performed using χ² tests or Fisher’s exact tests as appropriate.

Kaplan Meier analysis and Cox regression models were used to investigate the correlation between different groups and composite endpoint outcomes. Time to the composite endpoint was defined as the time to the first event, and a log-rank test was performed. Given the number of potential predictors and limited outcome events, Multivariable analysis were applied the rule of at least 10 events per variable to avoid model overfitting [17]. Refer to previous studies [13, 18], a core adjustment model was first built using pre-specified covariates—age, estimated glomerular filtration rate (eGFR), and left ventricular maximum wall thickness (LVMWT)—selected for their clinical relevance to disease natural history [4, 5]. We then employed forward stepwise selection to incorporate variables from univariate analysis in order of statistical significance (based on Wald statistics), including those with *p* < 0.1. Hypertension was selected as an indicator following this process. To assess multicollinearity, variance inflation factors (VIF) were calculated for each variable (see Supplementary Fig. 1).

For the observed missing data (QRS interval: *n* = 4, 4.8%; QRS axis: *n* = 4, 4.8%; QTc: *n* = 4, 4.8%), multiple imputation was applied to account for these gaps, generating 10 imputed datasets. Cox models were fitted to each imputed dataset, and results were pooled using Rubin’s rules to obtain robust estimates (Supplementary Table 2).

Statistical significance was set at *p* < 0.05. Analysis were conducted using SPSS (version 23.0; IBM Corp) and R (version 4.4.3; R Foundation).

## Results

The study included 83 FD patients (median age: 54 years, IQR: 42–62; 34.9% female). Baseline characteristics are summarized in Table 1. Most patients (79.5%, *n* = 66) received enzyme replacement therapy (ERT), and of this population, 29 patients (63.0% of those who had not received prior ERT) started ERT during follow-up.

**Table 1:** Baseline characteristics of the study population stratified by cardiac and renal involvement patterns

Clinical characteristic	Overall	non-affected	cardiac-only	renal-only	co-affected	*p*-value
Number	83	20	20	15	28	
Age at initial ECG (years)	54(42–62)	42(36–52)	58(54–66)	42(32–51)	55(43–63)	0.002
Female	29(35)	11(55)	7(35)	4(27)	7(25)	0.182
BMI	24(22–26)	24 (22–25)	24(23–27)	23(22–26)	22(21–24)	0.106
Clinical symptoms						
Chest pain or tightness	23(28)	1(5)	8(40)	4(27)	10(36)	0.037
Shortness of breath or palpitation	30(36)	3(15)	9(45)	6(40)	12(43)	0.139
Syncope or presyncope	8(10)	0(0)	3(15)	1(7)	4(14)	0.288
Hypertension	38(46)	4(20)	10(50)	6(40)	18(64)	0.022
Diabetes	9(11)	1(5)	2(10)	2(13)	4(14)	0.811
Cerebrovascular accident	5(6)	0(0)	1(5)	1(7)	3(11)	0.518
Coronary artery disease	7(8)	1(5)	3(15)	0(0)	3(11)	0.469
NYHA ≥ II	40(48)	2(10)	13(65)	3(20)	22(79)	< 0.001
Renal transplant	11(13)	0(0)	0(0)	3(20)	8(29)	0.003
Heart transplant	2(2)	0(0)	1(5)	0(0)	1(4)	1.000
ICD/PM	12(14)	1(5)	4(20)	0(0)	7(25)	0.070
Receiving ERT at baseline	37(45)	6(30)	9(45)	7(47)	15(54)	0.475
ERT initiated during study	29(35)	6(30)	6(30)	7(47)	10(36)	0.735
Phenotype						
Classical	32(39)	6(30)	3(15)	7(47)	16(57)	
Other	39(47)	10(50)	16(80)	5(33)	8(29)	
Not identified	12(14)	4(20)	1(5)	3(20)	4(14)	

Values are median (IQR) or n (%)

ERT = enzyme replacement therapy; ICD = implantable cardioverter-defibrillator; PM = pacemaker

Based on predefined heart/kidney damage criteria, patients were categorized in four groups: non-affected (24.1%, *n* = 20), cardiac-only (24.1%, *n* = 20), renal-only (18.1%, *n* = 15), or co-affected (33.7%, *n* = 28). Compared to the non-affected group, more male patients were observed in the heart and/or kidney involvement group. Patients in the cardiac-only and co-affected groups were notably older, with median ages of 58 and 55, respectively. A higher prevalence of chest pain or tightness was reported in these groups (40.0% and 35.7%). Functional impairment, defined as NYHA class ≥ II, was most common in the co-affected group (78.6%), followed by the cardiac-only group (65.0%). The burden of hypertension also varied considerably across groups, with the highest prevalence in the co-affected cohort (64.3%). In contrast, no significant differences were found for gender, BMI, symptoms of shortness of breath, syncope, diabetes, cerebrovascular accident, coronary artery disease, or ERT treatment status (*p* > 0.05 for all). The distribution of the classical phenotype was 38.6% in the overall cohort, with the highest prevalence in the co-affected group (57.1%).

Echocardiographic findings, Troponin I (TNI), brain natriuretic peptide (BNP), and eGFR for each group are detailed in Table 2. The echocardiographic and biomarker analysis revealed significant differences across groups for most parameters (*p* < 0.05), except for AO (Aortic Root) and LVEDD (Left Ventricular End-diastolic Diameter). Cardiac structural abnormalities, including increased LAVi, IVSD (Interventricular Septum Diastole), and LVMWT, were most pronounced in the cardiac-only and co-affected groups. Additionally, biomarkers TNI and BNP were markedly elevated in these groups. Renal function assessed by eGFR and overt proteinuria, was notably worse in the renal-only and co-affected groups. These findings highlight substantial cardiac and renal structural and functional alterations, particularly in patients with multi-organ involvement.

**Table 2:** Echocardiographic and biomarker characteristics of the population stratified by cardiac and renal involvement patterns

Variables	Overall	non-affected	cardiac-only	renal-only	co-affected	*p*-value
AO, mm	32(30–34)	31(31–33)	33(29–35)	31(30–31)	34(32–35)	0.059
LAVi, mL/m2	25(20–35)	20 (17–27)	27(20–43)	19(16–23)	30(21–40)	< 0.001
IVSD, mm	13(10–17)	9(9–10)	16(13–20)	10(9–11)	16(13–21)	< 0.001
LVMWT, mm	11(9–14)	9(8–10)	13(12–17)	9(8–10)	14(12–17)	< 0.001
LVEDD, mm	46(43–50)	45(42–47)	45(43–49)	49(45–52)	47(43–56)	0.201
LVESD, mm	29(27–32)	28(26–31)	29(25–31)	30(28–34)	32(27–45)	0.029
RVWT, mm	4 (4–6)	4 (4–6)	5 (4–8)	4 (4–5)	6 (4–8)	0.003
TAPSE, mm	21(20–23)	22 (20–24)	20(19–22)	23 (20–25)	20(19–23)	0.021
PASP, mmHg	20(18–28)	21(18–27)	20 (16–28)	25(20–34)	30 (24–33)	< 0.001
FS,%	36(32–40)	36(35–40)	38(32–43)	39(36–49)	32(23–36)	0.005
LVEF,%	65(60–70)	67(65–71)	64(59–72)	66(61–69)	61(46–66)	0.020
E/A ratio	1.1 (0.9–1.5)	1.4 (1.1–1.8)	1.0(0.8–1.3)	1.1 (0.9–1.5)	0.9(0.8–1.1)	0.007
Troponin I, ng/mL	0.050(0.008–0.247)	0.003(0.001–0.010)	0.067(0.015–0.436)	0.008(0.004–0.025)	0.222(0.075–0.406)	< 0.001
BNP, pg/mL	99(30–528)	20(10–32)	169(71–645)	60(36–131)	834(146–2734)	< 0.001
eGFR, mL/min/1.73 m^2^	79.3(45.7–99.2)	107.2(103.0-122.1)	92.3(90.4–99.2)	68.7(35.7-104.8)	44.8(24.2–54.3)	< 0.001
Overt proteinuria	27(33)	0(0)	0(0)	9(60)	18(64)	< 0.001

Values are median (IQR) or n (%)

AO = Aortic Root; LAVi = left atrial volume indexed(calculated on the basis of the ellipsoid formula); LVEDD = left ventricular end-diastolic diameter; IVSD = Interventricular Septum Diastole; LVEF = left ventricular ejection fraction; FS = Fractional Shortening; LVESD = left ventricular end-systolic diameter; RVWT = right ventricular wall thickness; TAPSE = tricuspid annular plane systolic excursion.LVMWT = left ventricular maximum wall thickness; PASP = pulmonary artery systolic pressure; BNP = B-type natriuretic peptide; eGFR = Estimated glomerular filtration rate; Overt proteinuria: Defined by values of > 300 mg/24 h

We then analysed standard and ambulatory electrocardiograms in the study population (Table 3). The Fabry cohort demonstrated a significant arrhythmia burden, with notable intergroup differences. Sinus bradycardia was the most prevalent arrhythmia overall, particularly in patients with cardiac-only involvement. In contrast, those with combined cardiac and renal involvement showed a higher incidence of overall abnormal cardiac rhythm, especially ventricular arrhythmias (*p* < 0.05). Although PR intervals did not differ significantly, the combined-involvement group exhibited more pronounced electrocardiographic abnormalities, including left QRS axis deviation, prolonged QRS duration, and QTc prolongation. In contrast, any of the conduction disorders, including various types of atrioventricular block and bundle branch blocks, showed no significant differences (*p* > 0.05 for all).

**Table 3:** Electrocardiographic characteristics of the study population stratified by cardiac and renal involvement patterns

Variables	Overall	non-affected	cardiac-only	renal-only	co-affected	*p*-value
Number	83	20	20	15	28	
Arrhythmia Data						
Patients with abnormal rhythm	50(60)	8(40)	15(75)	6(40)	21(75)	0.017
Sinus bradycardia	21(25)	5(25)	9(45)	3(20)	4(14)	0.118
Sinus tachycardia	2(2)	1(5)	0(0)	0(0)	1(4)	1.000
Premature atrial contraction	12(14)	2(10)	4(20)	1(7)	5(18)	0.669
Ectopic atrial rhythm	6(7)	1(5)	2(10)	0(0)	3(11)	0.715
Atrial fibrillation/Atrial flutter	7(8)	2(10)	3(15)	0(0)	2(7)	0.565
Ventricular tachycardia	3(4)	0(0)	2(10)	0(0)	3(11)	0.352
Premature ventricular beats	12(14)	0(0)	2(10)	2(13)	8(29)	0.032
Conduction Data						
Right bundle branch block	15(18)	2(10)	5(20)	2(13)	6(21)	0.633
Left bundle branch block	2(2)	0(0)	1(5)	0(0)	1(4)	1.000
Intraventricular block	5(6)	1(5)	1(5)	0(0)	3(11)	0.713
First-degree AVB/Second-degree type 1 AVB	8(10)	4(20)	1(5)	1(7)	2(7)	0.473
Second-degree type 2 AVB/Third-degree AVB	4(5)	1(5)	1(5)	0(0)	2(7)	0.905
Left ventricular high voltage	24(29)	3(15)	9(45)	5(33)	7(25)	0.204
Electrocardiographic characteristics						
Heart rate, beats/min	68(61–78)	74(62–84)	64(58–74)	69(62–79)	65(56–70)	0.263
PR Interval, ms	154(131–171)	151(134–171)	162(151–179)	140(125–159)	145(120–157)	0.174
QRS axis,°	51(0–76)	70(53–77)	43(0–65)	64(28–77)	16(-28-69)	0.032
QRS Interval, ms	96(82–106)	92 (80–108)	107(100–112)	87(83–97)	144(109–177)	< 0.001
QTc, ms	432(414–463)	415(402–432)	442.5(422–461)	419(388–438)	455(439–495)	< 0.001

Values are median (IQR) or n(%)

**Table 4:** Univariable/multivariable Cox regression analysis for the cardiovascular composite endpoint

Characteristic	Univariate logistic		Multivariate logistic	
	Odds Ratio (95%CI)	*p*-value	Odds Ratio (95%CI)	*p*-value
Female	0.554 [0.200, 1.440]	0.240		
Age	1.075 [1.033, 1.126]	< 0.001	1.066 [1.017, 1.126]	0.010
BMI	1.208 [0.952, 1.556]	0.130		
Hypertension	4.235 [1.669, 11.365]	< 0.001	3.646 [1.190, 12.049]	0.030
eGFR	0.978 [0.962, 0.992]	< 0.001	0.992 [0.966, 1.003]	0.100
LVMWT	1.176 [1.050, 1.336]	0.010	1.217 [1.056, 1.423]	0.010
HR	1.003 [0.969, 1.037]	0.860		
QRS.axis	0.994 [0.985, 1.003]	0.200		
QRS.interval	1.020 [1.005, 1.038]	0.010		
QTc	1.030 [1.015, 1.048]	< 0.001		

**Fig. 1:**
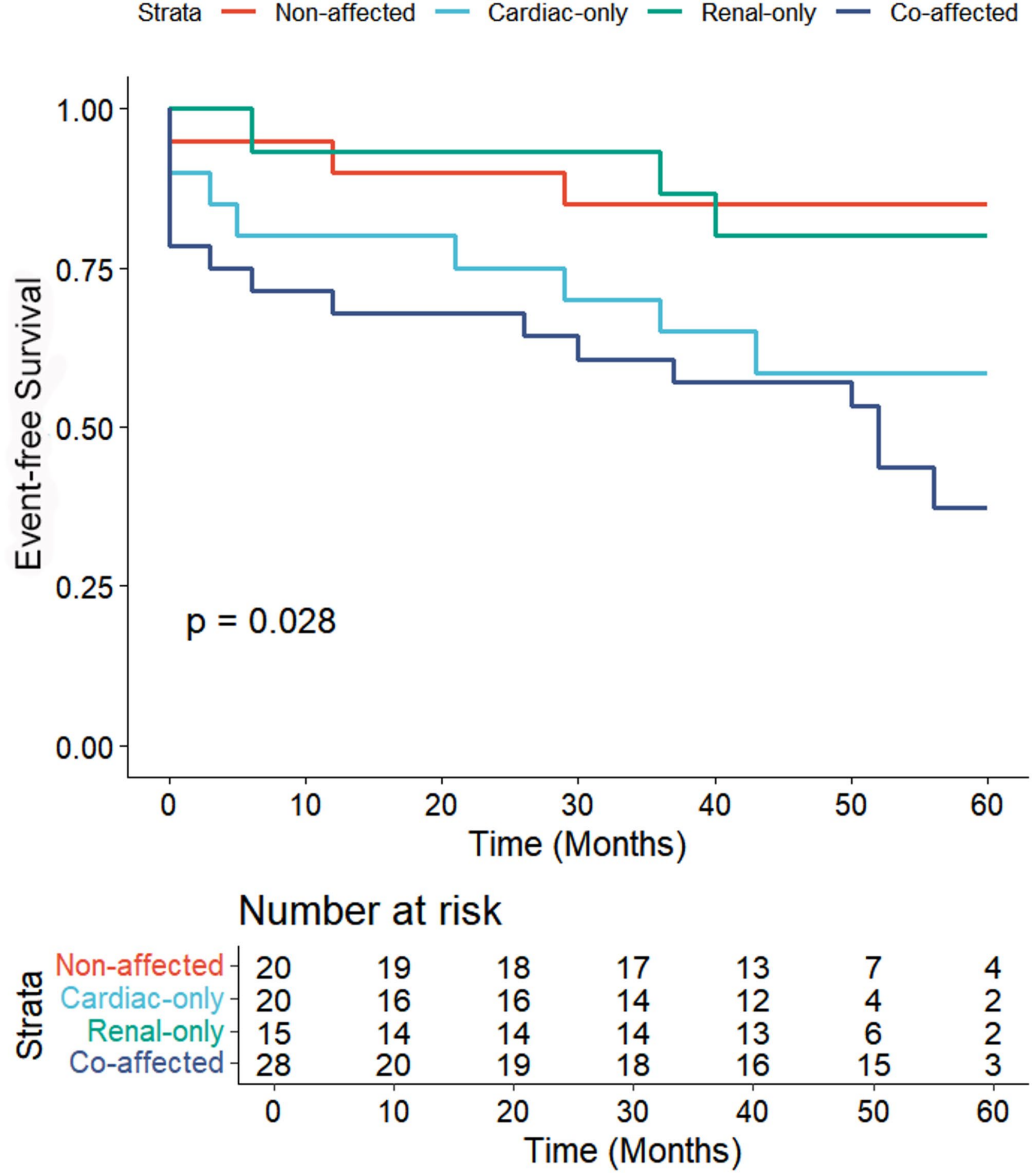
Cardiovascular outcomes according to the population stratified by cardiac and renal involvement patterns. Kaplan-Meier time-to-event curves for the composite endpoint of cardiovascular mortality, new-onset severe heart failure, incident atrial fibrillation, and significant arrhythmia requiring device implantation in patients with Fabry disease. The log-rank test revealed a statistically significant difference in event-free survival among the different patterns of cardiac and renal Involvement groups (*p* = 0.028) at 5 years

Kaplan-Meier analysis revealed a statistically significant difference in event-free survival among the four groups (Log-rank *p* = 0.028). The most favorable prognosis was found in the non-affected group, maintaining the highest survival probability throughout the 60-month follow-up. The renal-only group exhibited an intermediate survival curve, declining more gradually than the cardiac-involved groups (80.0% versus 60.0%). In contrast, both the cardiac-only and co-affected groups showed poorer outcomes, with a steeper decline in event-free survival probability over time (60.0% versus 85.0%;43.9% versus 85.0% log rank *p* < 0.001). The co-affected group consistently displayed the worst prognosis, with the earliest and most rapid event occurrence (see Fig.1).

Table 4 showed the correlates of the composite endpoint on univariable and multivariable Cox regression analyses. On univariable Cox regression analysis, eGFR (0.978 [0.962, 0.992] *p* < 0.001) and LVMWT(1.176 [1.050, 1.336] *p* = 0.010)expressed as continuous variables were significantly associated with the study endpoint. Conversely, the above parameters demonstrated differential associations with cardiovascular events, after correcting in multivariable Cox regression analyses (0.992 [0.966, 1.003] *p* = 0.100 and 1.217 [1.056, 1.423] *p* = 0.010; respectively).

## Discussion

In this study, a new classification system for patients with FD was developed based on comprehensive cardiac and renal assessments. This classification was used to systematically assess arrhythmia burden and prognostic outcomes across distinct clinical subgroups. This approach provides a robust framework for risk stratification in contemporary FD patient cohorts and provides valuable insights for clinical management.

Our study identified a subgroup without structural cardiac or renal impairment. As anticipated, these patients were younger and predominantly female (55%, *n* = 11), with few receiving disease-specific treatment (15%, *n* = 3). This subgroup represented a substantial proportion of our Fabry disease cohort, yet was characterised by high rates of missing baseline data and loss to follow-up (53.3%, *n* = 16). Although no significant abnormalities were detected in echocardiography or CMR, the frequent occurrence of left ventricular high voltage (15%, *n* = 3), sinus bradycardia (25%, *n* = 5), and atrial arrhythmias (25%, *n* = 5) suggests these features may represent early electrophysiological involvement. Previous studies have shown that in pre-hypertrophic FD, myocardial storage correlates with early electrocardiographic and morphological abnormalities [19, 20]. These early electrocardiographic and morphological abnormalities may hold significant clinical implications for this preclinical patient subgroup.

Cardiac involvement severity in FD is correlated with cardiovascular risk [13]. The high prevalence of Fabry disease-associated mutations among hypertrophic cardiomyopathy cases [21, 22] underscores the importance of systematic family screening for timely diagnosis. Our data demonstrate that structural cardiac involvement significantly increases arrhythmia incidence and is associated with prolonged QRS duration and QTc interval. This reveals the importance of rigorous arrhythmia monitoring and avoidance of QT-prolonging medications in affected patients. Furthermore, previous real-world evidence has suggested an association between advanced cardiac outcomes (especially AF) and late-onset variants [10]. In our study, we noted the special contribution of late-onset variants in cardiac involvement phenotypes, particularly the potential significance of c.639 + 919G >A (IVS4 + 919G >A) mutations in adverse cardiac outcomes, as previously reported [23]. Prospective studies targeting this specific location will be included in our future research plans.

Although the diagnostic criteria for Fabry renal involvement remain debated [24], CKD has been consistently recognized as a hallmark manifestation for FD [6]. Even modest elevations in albuminuria and small declines in eGFR were predictive of CKD progression and long-term adverse outcomes [25]. The risk of arrhythmia and cardiovascular events in these patients is still an interesting topic. In our observational study follow-up, 26.7% patients (*n* = 4) complained of chest pain, 40% patients (*n* = 6) complained of palpitations, shortness of breath and other suspected arrhythmia symptoms, however, malignant cardiac events were infrequently observed. Although our multivariable analysis did not identify a statistically significant correlation between eGFR and cardiovascular events, which was likely due to limited statistical power, emerging evidence demonstrates that renal dysfunction independently predicts adverse cardiovascular outcomes, particularly through its association with malignant arrhythmias and sudden cardiac death [26, 27]. A large-scale, prospective cohort study is required to definitively characterize the relationship between Fabry renal involvement and major cardiovascular events.

Co-affected group patients face a poorer prognosis compared to those with isolated organ manifestations. Impaired renal function is prevalent in chronic cardiovascular diseases, affecting approximately 40–60% of patients with congestive heart failure. Furthermore, cardiovascular events account for nearly 50% of deaths among all patients with CKD [28]. This collaborative pathophysiology establishes a cardiorenal vicious cycle through complex mechanisms [29, 30]. Traditionally, LVH is considered to be the strongest prognostic factor for Fabry cardiomyopathy, and also the main cardiac marker for disease-specific treatment and evaluation of treatment effect [31, 32]. In this study, LVMWT and hypertension were associated with cardiovascular events, which validates previous views and suggests the importance of blood pressure management in FD. Additionally, different characteristics of arrhythmias in the cardiac-only group and the co-affected group were observed. These findings suggested that attention should be paid to multi-organ system management and multidisciplinary joint diagnosis and treatment in the management of chronic Fabry disease.

## Conclusion

In Fabry disease, distinct patterns of cardiac and renal involvement demonstrate unique arrhythmia burdens and marked variations in cardiovascular events. LVMWT emerged as an independent predictor of cardiovascular events. These findings highlight the necessity for comprehensive risk stratification and multidisciplinary management strategies based on disease phenotype.

### Limitations

As a retrospective observational study, this study is not immune to sources of potential selection bias and unmeasured confounding bias, which may affect the generalisability of the findings [33]. The limited sample size precluded meaningful stratification according to the severity of cardiac or renal involvement. Furthermore, the relatively small cohort limits the robustness of subgroup (especially for the renal-only subgroup) and multivariable analyses. Our findings should be considered exploratory and hypothesis-generative in nature, requiring further validation through large-scale, prospective, multicentre study designs. Additional limitations include incomplete assessment of key clinical parameters: specifically, data regarding the initiation timing, duration, and compliance of enzyme replacement therapy were not fully captured, and arrhythmia surveillance was not standardised (e.g., Holter electrocardiography or implantable loop recorders). Additionally, although genotypic data are provided for all patients, further discussion is needed on detailed genotype-phenotype analysis.

## Supplementary Information

Below is the link to the electronic supplementary material.

Supplementary Material 1

Supplementary Material 2

Supplementary Material 3

## Data Availability

Data used is available from the corresponding author upon reasonable request. Consent was given for data sharing by participants.
