# Supplementary figures and images for: Arrhythmias and clinical outcomes in Fabry disease with cardiac and renal involvement
Source: Orphanet J Rare Dis. 2025 Nov 5;20:561. doi: 10.1186/s13023-025-04079-3 (PMC12587517; doi:10.1186/s13023-025-04079-3)

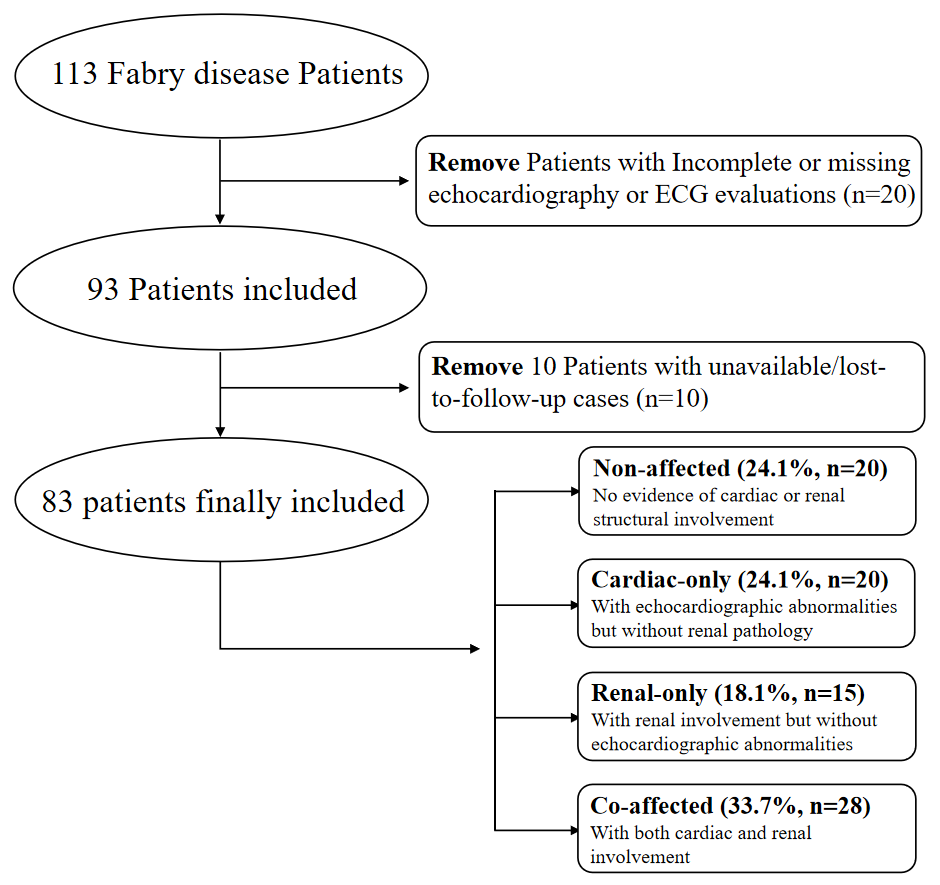

Supplement: Supplementary file 1 — Supplementary Material 1 [file 13023_2025_4079_MOESM1_ESM.tif]

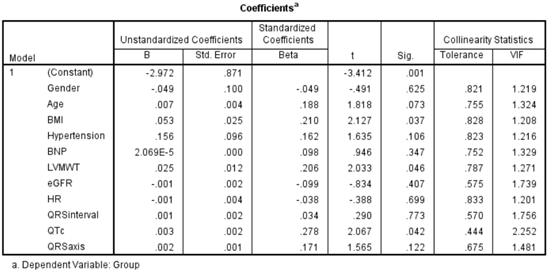

Supplement: Supplementary file 2 — Supplementary Material 2 [file 13023_2025_4079_MOESM2_ESM.tif]
