# Supplementary material for: Arrhythmias and clinical outcomes in Fabry disease with cardiac and renal involvement
Source: Orphanet J Rare Dis. 2025 Nov 5;20:561. doi: 10.1186/s13023-025-04079-3 (PMC12587517; doi:10.1186/s13023-025-04079-3)
Supplement: Supplementary file 3 — Supplementary Material 3 [file 13023_2025_4079_MOESM3_ESM.docx]

**Supplementary Table 1. Genotype characterisation of the study population.**

| **Mutations** | **N (%)** |
| --- | --- |
| **non-affected**  **c.639+919G>A**  **c.781G>T(p.Gly261Cys)**  **c.1218_1219delC>A(p.Ile407fs)**  **c.196G>C(p.Glu66Gln)**  **c.497T>C(p.Leu166Pro)**  **c.869T>C(p.Met290Thr)**  **c.881T>C(p.Leu294Ser)**  **c.196G>C(p.Glu66Gln)**  **c.369+102C>T**  **c.999+4A>G**  **Others**  **cardiac-only**  **c.639+919G>A**  **c.348del(p.lle117Phefs*4)**  **c.614C>A(p.Pro205His)**  **c.348delG(p.G116fs)**  **c.497T>C(p.Leu166Pro)**  **c.837G>T(p.Gln279His)**  **c.131G>A (p.Trp44Ter)**  **renal-only**  **c.1066C>G(p.Arg356Gly)**  **c.881T>C(p.Leu294Ser)**  **c.373C>T(p.His125Tyr)**  **c.868A>G(p.Met290Val)**  **c.272T>C(p.I91T)**  **c.838C>T(p.Gln280*)**  **c.1024C>T(p.Arg342*)**  **c.335G>A(p.Arg112His)**  **c.369+2T>G**  **c.1065C＞G**  **c.370-92G>C**  **c.370-680C>T**  **Others**  **co-affected**  **c.639+919G>A**  **c.869T>C(p.Met290Thr)**  **c.1197G>A(p.Trp399*)**  **c.838C>T(p.Gln280*)**  **c.695T>C(p.Ile232Thr)**  **c.348del(p.lle117Phefs*4)**  **c.2T>C(p.Met1?)**  **c.486G>C(p.Trp162Cys)**  **c.455A>G(p.Tyr152Cys)**  **c.902G>A(p.Arg301Gln)**  **c.803T>G(p.Leu268*)**  **c.1077_1120del(p.Gly360Argfs*5)**  **c.187T>C(p.Cys63Arg)**  **c.679C>(p.R227X)**  **c512G>A;p.Gly171Asp**  **c.902G>A(p.Arg301Gln)**  **c.187T>C(p.Cys63Arg)**  **c.334C>T**  **Others** | **8(40.0)**  **1 (5.0)**  **1 (5.0)**  **1 (5.0)**  **1 (5.0)**  **1 (5.0)**  **1 (5.0)**  **1 (5.0)**  **1 (5.0)**  **1 (5.0)**  **3(15.0)**  **13 (65.0)**  **2 (10.0)**  **1 (5.0)**  **1 (5.0)**  **1 (5.0)**  **1 (5.0)**  **1 (5.0)**  **2 (13.3)**  **1 (6.7)**  **1 (6.7)**  **1 (6.7)**  **1 (6.7)**  **1 (6.7)**  **1 (6.7)**  **1 (6.7)**  **1 (6.7)**  **1 (6.7)**  **1 (6.7)**  **1 (6.7)**  **2(13.3)**  **5 (17.9)**  **4 (14.3)**  **2 (7.1)**  **1 (3.6)**  **1 (3.6)**  **1 (3.6)**  **1 (3.6)**  **1 (3.6)**  **1 (3.6)**  **1 (3.6)**  **1 (3.6)**  **1 (3.6)**  **1 (3.6)**  **1 (3.6)**  **1 (3.6)**  **1 (3.6)**  **1 (3.6)**  **1 (3.6)**  **2(7.1)** |
